# Failure of the first step of two-stage revision due to polymicrobial prosthetic joint infection of the hip

**DOI:** 10.1007/s10195-016-0417-8

**Published:** 2016-07-07

**Authors:** Svetlana Bozhkova, Rashid Tikhilov, Dmitry Labutin, Alexey Denisov, Igor Shubnyakov, Vadim Razorenov, Vasilii Artyukh, Anna Rukina

**Affiliations:** 1 Vreden Russian Research Institute of Traumatology and Orthopaedics, Akademika Baikova Str., 8, 195427 St. Petersburg, Russian Federation; 2 Mechnikov North-Western State Medical University, St. Petersburg, Russian Federation

**Keywords:** Prosthetic joint infection, Two-stage revision, Polymicrobial PJI

## Abstract

**Background:**

The unsuccessful treatment of prosthetic joint infection (PJI) with two-stage revision leads to infection recurrence. The objectives of the study were to assess the clinical and demographic characteristics of patients with polymicrobial PJI, and to evaluate the role of the microbial profile involved in PJI in the risk of infection recurrence after the first step of two-stage revision surgery.

**Materials and methods:**

A retrospective analysis of 189 cases of culture-positive PJI following total hip replacement over a 5-year period was performed. The demographic characteristics of patients, clinical symptoms, microbiology cultures of intraoperative biopsies, laboratory values of C-reactive protein (CRP), white blood cell count and erythrocyte sedimentation rate were analyzed. Patients were divided into two groups—135 with monomicrobial and 54 with polymicrobial infection.

**Results:**

Of all patients, 68.9 % in the monomicrobial and 83.3 % in the polymicrobial group had a body mass index >25 kg/m^2^ (*p* = 0.05). The median CRP values were 5.7 mg/L (IQR 4.0–10.0 mg/L) in the monomicrobial compared to 8.8 mg/L (IQR 5.0–27 mg/L) in the polymicrobial group (*p* = 0.01). The percentage of successful outcomes was 27.8 % in patients with microbial associations (*p* < 0.0001). Gram-negative pathogens caused polymicrobial PJI in 61.5 % of cases with infection recurrence (OR 4.4; 95 % CI 1.18–16.37; *p* = 0.03).

**Conclusions:**

Overweight and obese patients or those with elevated CRP had a greater risk of polymicrobial PJI. They were predisposed to recurrence of infection after the first step of two-stage revision. An unsuccessful outcome was more likely in cases with polymicrobial infection compared to those with monomicrobial infection. In addition, the presence of multidrug-resistant strains of Gram-negative bacteria substantially increased the risk of PJI treatment being unsuccessful.

**Level of evidence:**

Level III, therapeutic study.

## Introduction

Prosthetic joint infection represents one of the most serious complications of total hip and knee arthroplasty. There have been vast improvements in operative techniques, quality of implant material and algorithms of antimicrobial therapy to prevent the onset of infection after surgery. Even though these measures appear adequate, development of PJI after total hip replacement is a clinically important issue. Its prevalence rate is estimated to be approximately 1.5 % with a mortality rate of 4 % within 90 days of the postoperative period [1]. The demand for joint arthroplasty is projected to grow in the coming years, thus the number of PJI cases is expected to increase [2].

Management of PJI includes several surgical procedures such as debridement and retention strategy, resection arthroplasty, arthrodesis, one-stage or two-stage exchange strategy combined with prolonged antimicrobial therapy and amputation of the lower limb in selected cases as a measure of last resort [3]. While most of these methods proved to be effective in the eradication of the infection, several studies demonstrated that two-stage revision protocol has a higher success rate ranging from 75−93 % [1, 4–9]. Therefore, this technique is currently considered as the gold standard in many countries [10].

The unsuccessful treatment of PJI with two-stage revision surgery leads to the recurrence of infection. This complication is usually associated with several risk factors such as age, overweight, comorbid conditions, alcohol abuse and the presence of pathogens resistant to antibiotics [11, 12]. Gram-positive cocci are the most common causative agents in PJI. They are identified in 84 % of cases, whereas the majority of them are *Staphylococcus aureus* and coagulase-negative staphylococci (CNS) [9]. At the same time, several studies report that Gram-negative bacteria are present in 11.5 [13, 14] and 15 % [13, 14] of PJI cases. These microorganisms are often associated with polymicrobial infections with a resistance to conventional antibiotic therapy. *Pseudomonas aeruginosa*, *Escherichia coli* and *Klebsiella pneumoniae* are frequent causative agents in this group of periprosthetic infections [15, 16]. Relapse of infection after two-stage exchange might be associated with Gram-negative bacteria. Successful outcomes of this strategy are reported to be less optimal and appear in 52 % of cases [13]. Collectively, all these factors make therapy of periprosthetic infection a challenging task.

Most studies on PJI therapy with two-stage exchange present observations from the follow-up period when the surgical procedure is completed. The unsuccessful outcome of this treatment leading to recurrent infections might also occur after the first step of two-stage exchange [17]. Therefore, information about the outcomes of this step is required to improve clinical recommendations for treatment of PJI.

The objectives of our study were (1) to assess the clinical and demographic characteristics of patients with polymicrobial PJI, and to (2) evaluate the role of the microbial profile involved in PJI in the risk of infection recurrence after the first step of two-stage revision surgery.

## Materials and methods

We collected and retrospectively reviewed 189 cases of culture-positive PJIs following total hip replacement. All data about patients were gathered from the medical records after approval by the Institutional Review Board.

This study covered a period of 5 years from 2008−2012. Among the patients, there were 92 males and 97 females with an overall median age of 57 years. The cohort comprised 144 cases of PJI after primary total hip arthroplasty (THA) and 45 cases after hip replacement revision surgery without any prior bone or joint infections. In all cases PJI was unilateral. All patients were treated by the three staff surgeons at the department of purulent infections at our institution.

The diagnosis of the hip PJI was confirmed by the presence of acute joint pain, a sinus tract communicating with the implant and wound dehiscence. Apart from the local symptoms, two or more positive microbiology cultures of synovial fluid aspirates or intraoperative tissue biopsies were taken into account. Laboratory parameters such as C-reactive protein (CRP), white blood cell count (WBC) and erythrocyte sedimentation rate (ESR) were also considered. In addition, the period before clinical manifestation of infection and its duration were evaluated.

All patients underwent the first step of two-stage revision which universally involves several surgical modalities such as the removal of a hip implant, debridement of infected periprosthetic tissues and subsequent insertion of a non-articulating or articulating spacer.

The initial procedure included evaluation of tissue viability in the affected area by the operating surgeon. Purulent tissues were exposed and visualized after an exploratory incision alone of the postoperative scar over the site of the implant. Up to five biopsy specimens of infected tissues and modular components of the implant were collected from each patient into sterile containers and transferred for microbiological analysis.

The next step of the procedure included ultrasonic-assisted debridement of infected tissues with the removal of the necrotic bone. Upon thorough debridement of the affected area, static spacers containing antibiotic-loaded bone cement, or articulating spacers with a metal femoral component were inserted. Bone cement (DePuy^*®*^ CMW 1 gentamicin) contained gentamycin with the addition of 2–4 g vancomycin per 40 g of material. Finally, the wound was closed with sutures following insertion of 3–4 drains with active suction. In some cases, coxofemoral immobilization was applied for 3 months after the operation, particularly in patients with high risk for dislocation of the affected hip joint.

During the postoperative period, all patients received intravenous antibiotic therapy for a period of 2 weeks followed by 4–6 weeks of oral therapy. The initial antibiotic regimen included combinations of vancomycin with beta-lactam antibiotics or quinolones; alternatively, beta-lactam antibiotics with quinolones or aminoglycosides. After evaluation of intraoperative biopsy cultures, the therapy was corrected in accordance with the antimicrobial sensitivity of identified pathogens.

Microorganisms were isolated from homogenized intraoperative tissue biopsies and biofilms from the surface of the removed implants after sonication. Microbial species were identified by the staff microbiologist from cultures with the use of selective media and biochemical test panels. Patients were divided into two groups according to the microbiology reports. The group with monomicrobial infection comprised 135 patients with only one type of identified microbial species, while the polymicrobial group included 54 patients with the presence of at least two or more different species of bacteria.

The outcome of the first step of the two-stage procedure was defined as successful when patients hospitalized for reimplantation had no recurrent infection. The outcome was interpreted as unsuccessful when inflammatory signs remained or reappeared during the period between the first step and reimplantation. These signs included the presence of acute inflammation with high levels of serum CRP, development of a sinus tract and relapse or reinfection, depending on the isolated microorganisms. In all cases of infection recurrence, the old spacer was replaced with a new one. Each group of patients with either monomicrobial or polymicrobial infection was further divided according to the outcomes into categories with or without infection recurrence.

Continuous variables are presented as medians with interquartile ranges (IQR). They were assessed for normality with D’Agostino-Pearson test and compared using the nonparametric Mann–Whitney *U* test. Categorical data are presented as counts and proportions, which were analyzed with Fisher’s exact test. The association between clinical factors and successful outcomes of surgery is shown as odds ratios (OR) with 95 % CI. Reported *p* values are two-tailed. A *p* value <0.05 was considered significant. All statistical tests were performed with GraphPad Prism 6.0 (CA, USA).

## Results

Demographic characteristics of the patients, clinical presentation of PJI and laboratory findings are reported in Table 1. They showed no considerable difference between groups with monomicrobial and polymicrobial infection except for body mass index (BMI) and serum CRP levels. The majority of patients were overweight or obese (73 %, *n* = 138). The median BMI was 26 kg/m^2^ (IQR 24–28 kg/m^2^) in the monomicrobial group compared to 28 kg/m^2^ (IQR 26–30 kg/m^2^) in the polymicrobial group (*p* = 0.01). Of all patients, 68.9 % in the monomicrobial and 83.3 % in the polymicrobial group had a BMI >25 kg/m^2^ (*p* = 0.05). Variation of serum CRP levels reached significance with median values of 5.7 mg/L (IQR 4.0–10.0 mg/L) in the monomicrobial group compared to 8.8 mg/L (IQR 5.0–27 mg/L) in the polymicrobial group (*p* = 0.01).

**Table 1:** Demographic, clinical and laboratory characteristics of patients with monomicrobial and polymicrobial PJI

Variable	Monomicrobial infection (*n* = 135)	Polymicrobial infection (*n* = 54)	*p* value
Age, years	57 (49–67)	57 (44–69)	0.84
WBC × 10^3^/μL	8.1 (6.2–9.9)	7.3 (5.5–9.1)	0.1
BMI, kg/m^2^	26 (24–28)	28 (26–30)	0.01
≤25, *n*	42 (31.1 %)	9 (16.7 %)	0.05
>25, *n*	93 (68.9 %)	45 (83.3 %)	
ESR, mm/h	25 (15–40)	26 (15–49)	0.64
≤30, *n*	85 (63.0 %)	31 (57.4 %)	0.51
>30, *n*	50 (37.0 %)	23 (42.6 %)	
CRP, mg/L	5.7 (4.0–10.0)	8.8 (5.0–27)	0.01
≤10, *n*	101(74.8 %)	31 (57.4 %)	0.02
>10, *n*	34 (25.2 %)	23 (42.6 %)	
PJI after prior revision surgery, *n*	34 (25.1 %)	11 (20.4 %)	0.57
Manifestation of infection, days	365 (90–1500)	600 (120–1575)	0.76
Pain, *n*	134 (99.3 %)	54 (100 %)	1.0
Sinus tract, *n*	100 (74.1 %)	46 (85.2 %)	0.13
Wound dehiscence, *n*	1 (0.7 %)	0	1.0
Duration of infection, days	150 (60–300)	165 (60–400)	0.47
Duration of surgery, min	190 (160–240)	200 (171–230)	0.62
Blood loss, mL	800 (600–1200)	900 (600–1200)	0.71
Drainage blood loss, mL	250 (140–350)	300 (200–300)	0.70
With articulating spacer, *n*	73 (54.1)	24 (44.4)	0.26
Concomitant pathology			
Cardiovascular pathology, *n*	99 (73.3 %)	36 (66.7 %)	0.37
Diabetes mellitus, *n*	27 (20.0 %)	11 (20.4 %)	1.0
COPD, *n*	24 (17.8 %)	8 (14.8 %)	0.68
Indication for THA			
Osteoarthritis, *n*	80 (59.3 %)	39 (72.2 %)	0.13
Secondary osteoarthritis with rheumatoid arthritis, *n*	12 (8.9 %)	2 (3.7 %)	0.36
Femoral head fracture, *n*	19 (14.1 %)	5 (9.3 %)	0.47
Avascular necrosis of the femoral head, *n*	24 (17.8 %)	8 (14.8 %)	0.68

All continuous variables are presented as medians with IQR

**Fig. 1:**
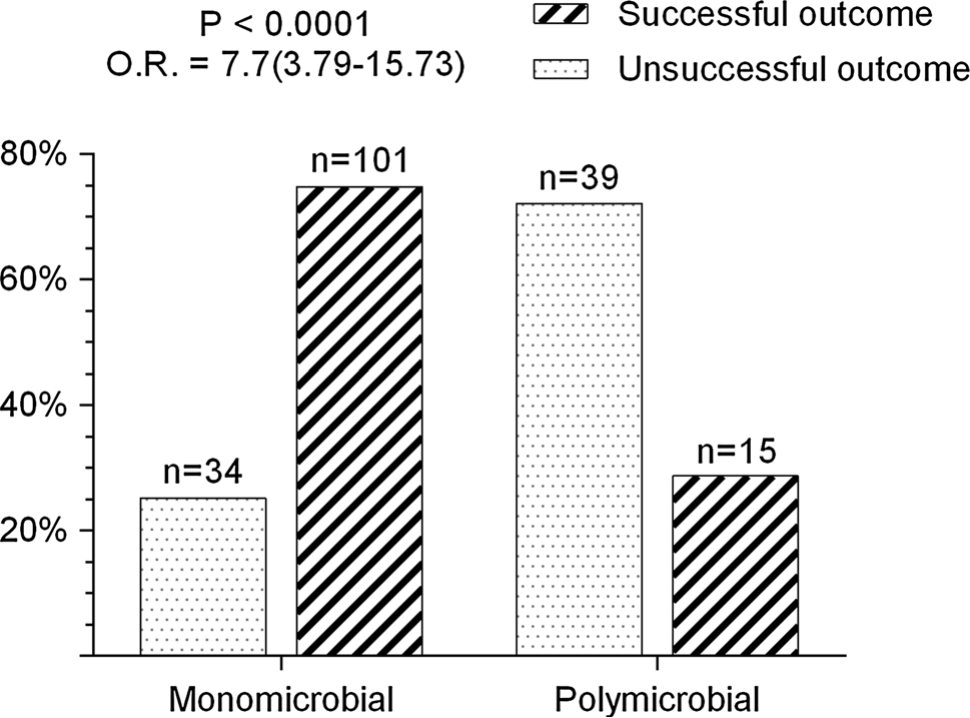
Percentage of cases with successful and unsuccessful outcomes in monomicrobial and polymicrobial PJI

According to the widely accepted classification of PJI [18], 27.4 % of monomicrobial and 20.4 % of polymicrobial infections in our cohort were early and occurred within 3 months after THA; 23.7 and 25.9 % were delayed and occurred between 3 and 12 months after surgery, respectively. Approximately half of all cases represented late PJI which occurred at ≥12 months after THA. Polymicrobial infection was identified in 28.6 % (*n* = 54) of all culture-positive PJIs (*n* = 189). Gram-positive pathogens were predominant in both groups (*p* = 0.02). *S. aureus* accounted for 52.6 % of isolates in the monomicrobial group (*p* = 0.0002) (Table 2). Of all *S. aureus* isolates, 8.5 and 20.6 % were methicillin-resistant in the monomicrobial and polymicrobial groups, respectively. Methicillin-resistant *Staphylococcus epidermidis* (MRSE) was present in 24.3 and 31.6 % of all *S. epidermidis* isolates in both groups. Overall, the frequency of CNS isolates in the polymicrobial group was the highest (34.5 %) among all bacterial strains. The percentage of successful outcomes after the first step of the two-stage procedure was considerably higher (74.8 %, *n* = 101) in patients with monomicrobial infection, compared to only 27.8 % (*n* = 15) in the polymicrobial group (*p* < 0.0001) (Fig. 1). Cases with microbial associations were more likely to have an unsuccessful outcome (OR 7.7; 95 % CI 3.79–15.73).

**Table 2:** Variety of pathogens isolated from patients with monomicrobial and polymicrobial PJI

Pathogens	Monomicrobial infection *n* (%)	Polymicrobial infection *n* (%)	*p* value
All Gram-positive	116 (85.9)	101 (74.3)	0.02
*S. aureus*/MRSA	71/6* (52.5/8.5*)	34/7* (25/20.6*)	<0.01/0.11
*S. epidermidis*/MRSE	37/9** (20.1/24.3**)	38/12** (27.9/31.6**)	0.15/1.0
Other CNS	4 (3.0)	9 (6.6)	1.0
*Enterococcus* sp.	6 (4.4)	11 (8.1)	1.0
*Streptoccus* spp.	4 (3.0)	6 (4.4)	1.0
*Propionibacterium acnes*	2 (1.5)	6 (4.4)	0.71
*Corynebacterium* spp	2 (1.5)	3 (2.2)	1.0
Other Gram-positive	4 (3.0)	3 (2.2)	0.41
All Gram-negative	19 (14.1)	35 (25.7)	0.02
*Enterobacteriaceae*	7 (5.1)	13 (9.6)	0.16
*Escherichia coli*	3 (2.2)	6 (4.4)	0.31
*Klebsiella pneumoniae*	1 (0.7)	2 (1.5)	0.6
*Klebsiella pneumoniae* (ESBLs)	1 (0.7)	3 (2.2)	0.35
*Enterobacter cloacae*	2 (1.5)	2 (1.5)	1.0
*Serratia marcescens*	2 (1.5)	1 (0.7)	1.0
*Providencia* sp.	0	1 (0.7)	0.47
*Aeromonas* sp.	0	1 (0.7)	0.47
*Alcaligenes* sp.	0	1 (0.7)	0.47
*Acinetobacter* sp.	3 (2.2)	10 (7.4)	0.045
*Pseudomonas* *aeruginosa*	5 (3.7)	7 (5.1)	0.56
*Stenotrophomonas*	1 (0.7)	1 (0.7)	1.0
*Actinobacillus*	1 (0.7)	0	1.0
*Candida* sp.	0	1 (0.7)	0.47
Total	135	136	

Values are presented as the number of isolates. Data were analyzed with Fisher’s exact test

* Number (%) of all *S.* *aureus* in the group; ** number (%) of all *S.* *epidermidis* in the group

**Fig. 2:**
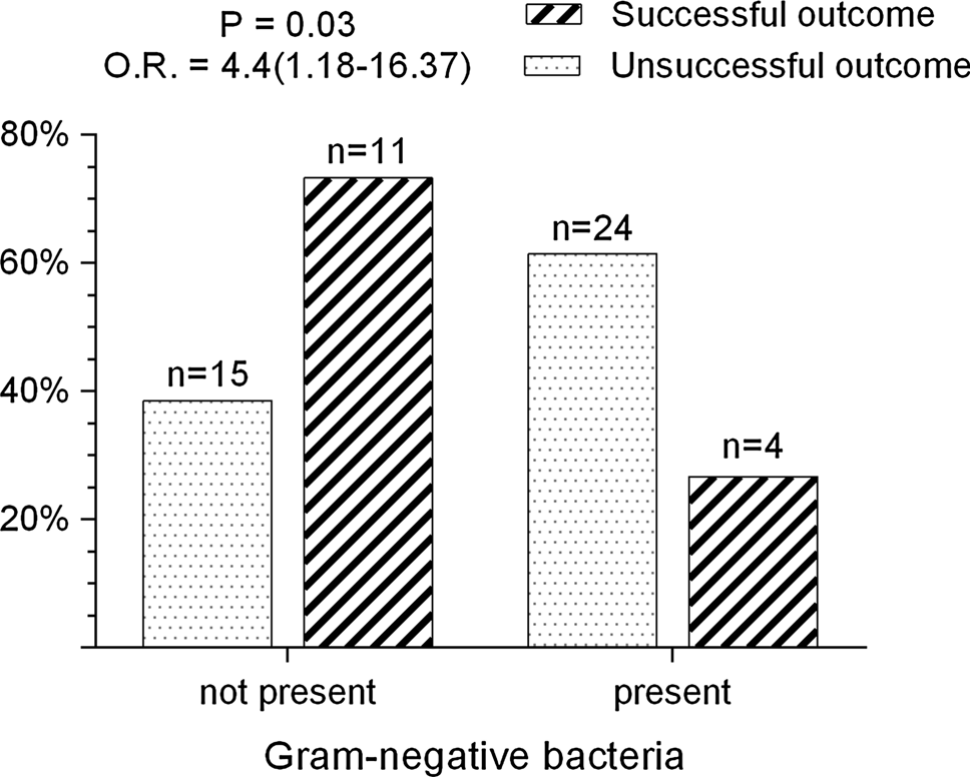
Percentage of cases with successful and unsuccessful outcomes in polymicrobial PJI depending on the presence of Gram-negative pathogens in microbial associations

Gram-negative pathogens were frequently identified in associations (*p* = 0.02). They accounted for 14.1 and 25.7 % of isolates in the monomicrobial and polymicrobial groups, respectively. Non-fermenting bacteria prevailed among Gram-negative strains. *Acinetobacter sp.* and *P. aeruginosa* were identified in 7.4 % (*p* = 0.05) and 5.1 % (*p* = 0.56) of isolates, respectively. The proportion of polymicrobial PJI caused by Gram-negative pathogens was 61.5 % in patients with recurrent infection and only 26.7 % in patients with treatment success (*p* = 0.03) (Fig. 2). The presence of Gram-negative pathogens was associated with infection recurrence (OR 4.4; 95 % CI 1.18–16.37).

## Discussion

The gold standard for PJI management is two-stage revision surgery with subsequent local and systemic antibiotic therapy [10]. Although many studies focus on the outcomes of the final step of the exchange procedure, unsuccessful outcome often occurs after the initial step when an antibiotic-laden spacer is inserted [17]. This study attempts to elucidate factors leading to infection recurrence after the first step of two-stage revision.

International guidelines for diagnosis and treatment of PJI recommend several sensitive diagnostic markers such as serum CRP and ESR levels [3, 19]. We found that CRP levels were significantly increased in patients with polymicrobial infection reaching a median of 8.8 mg/L. This was in agreement with suggested prognostic criteria but below the diagnostic value of 10 mg/L for periprosthetic infection after THA [20–23]. The median ESR level was below the suggested value of 30 mm/h with no significant difference between study groups. We believe that the low activity of these inflammatory markers in our patients was due to the chronic state of PJI.

Concomitant somatic conditions are usually considered an important risk factor of PJI [11, 12]. In our study, the incidence of cardiovascular pathology and diabetes mellitus was high in both groups but polymicrobial PJI occurred regardless of these predisposing conditions. Although the majority of our patients were either overweight or obese with a BMI >25 kg/m^2^, 83.3 % (*n* = 45) of them had polymicrobial infection. Thus, these are the factors associated with the development of PJI which particularly increase the chance of polymicrobial infection [24]. Patients with a BMI >25 kg/m^2^ had a greater risk of infection caused by microbial associations (OR 2.3; CI 95 % 1.01–5.04).

In a case–control study, Berbari et al. [25] reported that patients who had arthroplasty prior to THA or total knee arthroplasty were under a great risk of PJI. As depicted in Table 1, the majority of our patients had PJI after primary THA. The proportion of these patients with infection recurrence after the first step of the two-stage procedure was higher than those with prior revision arthroplasty. Presumably, this happened due to the long duration of PJI, i.e., from onset until the removal of the prosthesis. The median time period was 150 (IQR 60–300) and 160 (IQR 60–400) days in the monomicrobial and polymicrobial groups, respectively.

In our study, polymicrobial infection was diagnosed in 28.6 % cases of PJI after THA which is close to the range of 19–37 % reported elsewhere [26, 27]. These authors also showed that polymicrobial PJIs occur more often in the early postoperative period. We did not observe any significant difference in manifestation time of infection between study groups. The median time period was 365 days for monomicrobial PJI and 600 days for polymicrobial infection (*p* = 0.76). The majority of our PJI cases were either delayed or late infections.

Polymicrobial infection is traditionally considered a risk factor for failure of one-stage revision in the management of PJI presenting a contraindication for this type of surgery [19, 28]. The cumulative probability of PJI treatment success with surgical revision reported by Marculescu et al. [26] was 63.8 % for cases with polymicrobial PJI and 72.8 % for those with monomicrobial infection. The authors showed that the 2-year survival rate without infection after the two-stage procedure was 83.9 and 77.7 % for monomicrobial and polymicrobial PJI, respectively. The difference was statistically insignificant due to the small number of observations (49 and 9 cases of monomicrobial and polymicrobial infection, respectively). Comparable rates were also reported by Wimmer et al. [29] in a cohort of 77 cases of total hip and knee arthroplasty, where 87.5 % of their patients with monomicrobial compared to 67.6 % with polymicrobial PJI were free of infection. In our study, the outcome was defined after the first step of the two-stage procedure. Although our results could not be directly compared to the above reports due to this discrepancy, we found a similar rate of success (74.8 %) in monomicrobial PJIs, but a lower (27.8 %) rate in cases with polymicrobial infection. Moreover, cases with microbial associations in our cohort were more likely to result in infection recurrence (OR 7.7; CI 95 %, 3.79–15.73).

Gram-positive bacteria were dominant in both study groups with *Staphylococcus sp.* being the most frequent pathogens (Table 2). Some authors indicate that the presence of methicillin-resistant strains predicts an unsuccessful outcome of PJI [11]. In our study, identification of these strains was not associated with unsuccessful outcomes of the first step of two-stage revision. In the group with monomicrobial infection, the proportion of methicillin-resistant strains in patients with unsuccessful and successful outcomes was 8.7 and 17.3 %, respectively. Similar findings were found in the polymicrobial group with 23.6 and 35.3 % of all staphylococci, respectively. This might be due to (a) incorporation of vancomycin into a gentamicin-loaded cement spacer, or (b) administration of empiric antimicrobial therapy that always included vancomycin combined with beta-lactams or quinolones in patients with negative cultures of aspirates or without any preoperative results. As a result, initial local and systemic antimicrobial therapy was effective against Gram-positive bacteria including both methicillin-sensitive and methicillin-resistant strains of staphylococci. As evident from our data, 79.3 % of patients with Gram-positive monomicrobial PJI had sustained remission of the infection.

In most cases, antibiotics effective against multidrug-resistant (MDR) strains of Gram-negative bacteria (extended-spectrum beta-lactamase [ESBL]-producing *K. pneumonia*, *Acinetobacter sp.* and *P. aeruginosa*) were administered only after isolation of these pathogens from intraoperative cultures of infected tissues and/or removed components of the implant. In addition, gentamicin which is released from bone cement has low activity against this type of pathogens. Therefore, ineffective initial therapy with local antibiotics present in cement spacers and systemic antimicrobial regimens might have led to an unsuccessful outcome of PJI. Of nine patients with monomicrobial PJI, caused by Gram-negative MDR strains, only one patient had sustained remission. Of 18 patients with polymicrobial infection, caused by microbial associations with MDR strains, the first step of the two-stage procedure was also successful in one patient.

Our study had several limitations. Firstly, this is a single-center study. Due to its retrospective nature, it was not always possible to collect a complete case history. In particular, other concomitant conditions that might be a risk for PJI or details regarding previous arthroplasties were not always available. Secondly, the initial antibiotic therapy in our cohort was varied. There were patients who received either empirical or causative therapy. Thirdly, there might have been slight deviations in the surgical technique of two-stage revision because not every case was managed by the same operating surgeon. Finally, we did not follow-up patients with an unsuccessful outcome after replacement of the old spacer with a new one. Considering the difficulty in collecting an appreciable number of cases with PJI for a study, the relatively large number of patients (*n* = 189) in our cohort should be sufficient to overcome potential bias.

In conclusion, polymicrobial infection was observed in 28.6 % of all PJI cases. Approximately half of all cases presented with late PJI which occurred at ≥12 months after THA. We found that overweight and obese patients or those with elevated CRP levels had a greater risk of polymicrobial PJI and hence they were predisposed to recurrence of infection. Cases that presented with polymicrobial infection were more likely to result in an unsuccessful outcome after surgery (72.2 %) compared to those with monomicrobial infection (25.2 %). In addition, the presence of MDR strains of Gram-negative bacteria substantially increased the risk of failure for this type of surgery in both study groups. Despite the fact that leading pathogens in both the monomicrobial and polymicrobial groups were staphylococci, cases of PJI where empirical antibacterial therapy was administered required local and systemic antimicrobial combinations that are active against MDR strains of Gram-negative bacteria. We conclude that it is necessary to perform further multicenter prospective studies on polymicrobial PJI in a larger cohort of patients in order to identify other risk factors and develop effective measures to reduce the chance of adverse outcomes.
